# Incomplete Recovery of Zebrafish Retina Following Cryoinjury

**DOI:** 10.3390/cells11081373

**Published:** 2022-04-18

**Authors:** Denisa Džulová, Dylan Lawless, Gaëtan G. Pinton, Nicole A. Renner, Daniel F. Schorderet

**Affiliations:** 1 Institute for Research in Ophthalmology, 1950 Sion, Switzerland; d.dzulova@gmail.com (D.D.); gaetan.pinton@gmail.com (G.G.P.); niki.renner@gmail.com (N.A.R.); 2 Global Health Institute, School of Life Sciences, École Polytechnique Fédérale de Lausanne, 1015 Lausanne, Switzerland; dylan.lawless@epfl.ch; 3 Faculty of Biology and Medicine, University of Lausanne, 1015 Lausanne, Switzerland

**Keywords:** retina, zebrafish, cryoinjury, regeneration

## Abstract

Zebrafish show an extraordinary potential for regeneration in several organs from fins to central nervous system. Most impressively, the outcome of an injury results in a near perfect regeneration and a full functional recovery. Indeed, among the various injury paradigms previously tested in the field of zebrafish retina regeneration, a perfect layered structure is observed after one month of recovery in most of the reported cases. In this study, we applied cryoinjury to the zebrafish eye. We show that retina exposed to this treatment for one second undergoes an acute damage affecting all retinal cell types, followed by a phase of limited tissue remodeling and regrowth. Surprisingly, zebrafish developed a persistent retinal dysplasia observable through 300 days post-injury. There is no indication of fibrosis during the regeneration period, contrary to the regeneration process after cryoinjury to the zebrafish cardiac ventricle. RNA sequencing analysis of injured retinas at different time points has uncovered enriched processes and a number of potential candidate genes. By means of this simple, time and cost-effective technique, we propose a zebrafish injury model that displays a unique inability to completely recover following focal retinal damage; an outcome that is unreported to our knowledge. Furthermore, RNA sequencing proved to be useful in identifying pathways, which may play a crucial role not only in the regeneration of the retina, but in the first initial step of regeneration, degeneration. We propose that this model may prove useful in comparative and translational studies to examine critical pathways for successful regeneration.

## 1. Introduction

The remarkable ability of teleost to regenerate following injury, including central nervous system (CNS) tissues, such as the retina and spinal cord is well known [1]. This suggests the alluring possibility that CNS regeneration may be attainable in humans through gene modulation techniques. Of critical importance is the understanding of the pathways and mechanisms underlying successful and, imperatively, unsuccessful regeneration in order to recreate the regeneration process for clinical applications. For this, RNA sequencing is a very useful tool in identifying potential genes undergoing differential expression in cryoinjured retinas, which may be crucial in retinal degeneration and regeneration processes.

It has previously been shown that neural regeneration in the adult zebrafish is dependent on the acute inflammatory response [2,3] and on the differentiation of Müller glia (MG) into neurons [4]. In mammals, it was first believed that the glial inflammatory response impedes regeneration and repair [5] by inducing reactive gliosis and gliotic scarring [6]. Recently, studies have shown the potential of adult mouse MG cells to re-enter the cell cycle after the overexpression of proliferating MG marker *Ascl1*, to induce proliferation and to generate limited retinal neurons to some extent [7]. However, in a more recent study, mammalian MG cells were found to be in dormant state due to the repression of the active Hippo signaling pathway [8]. Therefore, it appears that the heterogeneous mammalian glial response to injury includes an attempt to recreate differentiation cues and axonal pathfinding signals; however, this process is still hindered by obstructive molecular mechanisms. Thus, the goal of clinical intervention is to manipulate the post-injury environment in such a way that successful regeneration of neural tissue will proceed and have long lasting effects.

A variety of retinal injury models already exist in the zebrafish, including light-induced lesions, laser ablation, targeted cell ablation, thermal lesions, optic nerve crush and mechanical injury by needle [9,10,11,12,13,14,15,16,17,18]. One consistent hallmark of these injury models is the restoration of a complete retina with correct lamination, which is functionally integrated into the optic nerve. Perturbations from the original retina have been noted in these models, especially a failure to recreate the photoreceptor array pattern typically established during embryonic development [17,19].

Here, we present a novel cryoinjury zebrafish model, which is characterized by an initial coagulative necrotic period, followed by infiltration of inflammatory cells and MG to the lesion site. The injured retina then undergoes a recovery phase where MG are differentiated into new photoreceptors and incompletely repair the retina. Uniquely, this model results in a portion of the injury site with long-lasting, persistent gliosis similar to the gliosis observed in mammals. We propose that this method may prove useful to identify key genes that act to impede or enhance regeneration. This study outlines a suitable zebrafish model of retinal injury for studying not only retinal regeneration, but also retinal degeneration in humans.

## 2. Materials and Methods

### 2.1. Animal Statement

Wild-type AB fish (European Zebrafish Resource, Karlsruhe, Germany) or transgenic Tg(*gfap:GFP*)mi2001 fish (ZIRC, Eugene, OR, USA) expressing GFP in glial cells under a GFAP promoter were raised and kept under standard laboratory conditions, system water was maintained at 26.5 °C [20]. Zebrafish were euthanized in a 30.6 mM Tricaine (Sigma A5040) solution in a time-course study at 0 dpi, 3 dpi, 7 dpi, 30 dpi, 100 dpi and 300 dpi. Zebrafish were left in Tricaine solution for 10 min following cessation of opercular movement. All experiments were approved by the Veterinary Service of the Canton of Valais (Switzerland, VS-10).

### 2.2. Cryoinjury

Adult zebrafish measuring between 25 and 35 mm in length were anesthetized in a solution containing 30.6 mM tricaine (Sigma A5040) until respiration slowed, fish were unresponsive to tapping near the container and cessation of opercular movement has occurred. At this point, each fish was removed from the tricaine solution and placed on a sponge under a dissecting microscope. A probe (Milian 076257) was used to apply pressure to the anterior part of the right eye, thus rotating the eye and exposing the temporal sclera. Cryoinjury was performed by applying a second probe of approximately 0.5 mm in diameter (area of 0.39 mm^2^), pre-cooled in liquid nitrogen, which was held against the exposed sclera while applying gentle pressure for 1 s, and then removed. There was no puncture wound or physical injury performed with the probe itself; gentle pressure was sufficient to flatten the surface of the area under the probe to provide even application of the cryoprobe. During the application, formation of ice crystals was detected across the surface of the sclera originating at the site of probe contact. The ice resolved quickly after the probe was removed. Fish were left to recover from anesthesia in breeding tanks containing system water before being returned to system tanks. Fish were sacrificed at selected time points (0 dpi, 3 dpi, 7 dpi, 30 dpi, 100 dpi and 300 dpi) and eyes removed for analyses.

### 2.3. Retina Dissection

Fish were euthanized in a 30.6 mM tricaine (Sigma A5040) solution in a time-course study at 0 dpi, 3 dpi, 7 dpi, 30 dpi, 100 dpi and 300 dpi. Right eyes were dissected shortly thereafter. Briefly, under a dissecting microscope the conjunctiva was removed using forceps and the optic nerve and associated blood vessels were sectioned. The removed eye was placed in a drop of E3 media (for 60× solution in 2L: 34.8 g NaCl, 1.6 g KCl, 5.8 g CaCl_2_-2H_2_O, 9.78 g MgCl_2_-6H_2_O. Adjust pH to 7.2 with NaOH), and a small opening was cut in the cornea using scissors. The scissors were then used to cut a complete circle at the anterior edge of the iris. The cornea, iris and lens were gently extracted, leaving the eyecup and retina intact. For histological analysis, the eyes were fixed in 4% PFA (Fluka, 76420) for 2 h at 4 °C; cryoprotected by incubating in graded sucrose solutions, with a final solution concentration of 30% sucrose, at 4 °C. Eyes were embedded in cryomolds (Medite Service AG, 48-6314-00) using “Yazulla” medium: 30% albumin (ACROS Organics 400455000) and 3% gelatin (Fisher Chemicals 9000-70-8) in ultrapure water, frozen over dry ice and stored at −80 °C. Eyes were cryosectioned at 10 µm thickness, collected onto positively charged glass slides (ThermoFisher Scientific, J1800AMNZ) and stored at −20 °C.

Tissues used for RNA analysis were further dissected; the enucleated eyes were placed in a drop of RNA*later*™ (Ambion AM7024, Life Technologies) and the eye cup gently inverted to expose the retina. Using forceps, the retina was dissected away from the sclera and choroid vasculature trying to exclude retinal pigment epithelium (RPE). Isolated retinas were collected in RNA*later*™.

### 2.4. Histology

Hematoxylin and eosin (H & E) staining on sections from WT and cryoinjured fish was performed using standard techniques.

### 2.5. Progression and the Regression of the Lesion

In order to follow the extent of damage and the recovery, the size of the lesion through the recovery phase was evaluated using Tg(*gfap:GFP*)Mi2001 fish. This allowed for clear visualization of the MG, which are known to serve as the source of stem cells in the zebrafish retina. Dissections were performed as described above, except that after removing the lens and vitreous, the eye was cut in half leaving the temporal half, where the lesion was located, easily accessible for imaging. Images were taken using a Leica MZ15F (Leica, Weitzlar, Germany) dissecting microscope. For each image collected, a corresponding image was taken using a calibration slide.

### 2.6. Immunofluorescence

Slides of frozen sections from WT and cryoinjured fish were dried at 55 °C for 10 min before being incubated in phosphate-buffered saline (PBS) at room temperature (RT) for 10 min. Blocking was performed for 1 h at RT in a solution containing PBS, 2% normal goat serum (Sigma G9023), and 0.2% Triton-X100 (ACROS Organics, 327371000). Primary antibodies (Table S1) were diluted in blocking buffer and allowed to bind overnight at 4 °C. Excess primary antibody was removed by rinsing slides 3× with PBS. Slides were blocked again, using the same blocking buffer, and secondary antibodies (Table S1) diluted in blocking buffer were incubated on slides for 30 min at RT in dark. Excess secondary antibody was washed 3× with PBS, and slides were counterstained with DAPI (Molecular Probes D3571) diluted 1:333 in PBS for 10 min in the dark. Excess DAPI was washed off 3× with PBS. Slides were mounted using CitiFluor mounting media (Citifluor AF1-100).

For slides stained with PCNA antibody, an epitope retrieval step was added before the first blocking solution. Epitope retrieval was performed in 10 mM sodium citrate (pH 6) at 100 °C for 15 min, slides were then cooled for 10 min in a solution containing PBS and 0.05% Triton-X100. All other steps were as previously described. Slides were imaged on a Leica DM6000B (Leica, Weitzlar, Germany). All antibodies and their dilutions are listed in Table S1.

### 2.7. RNA Extraction

Six retinas were pooled to create a biological sample, for each time point 3 independent biological samples were used (n = 3 for statistical analysis). Control samples were taken from uninjured WT fish to avoid any systemic effects of a contralateral injury impacting gene expression. RNA extraction was carried out on homogenized samples using RNeasy Mini Kit with filter columns (Qiagen, Hilden, Germany, 74104) with no deviations to the manufacturer’s protocol. Briefly, retinal tissues were homogenized by shearing using a 26-gauge needle (Sterican) in RLT buffer containing β-mercaptoethanol (Applichem, A1108). Elution of RNA was performed using 30 µL of RNAse-free water. Digestion with DNAse was performed using DNase Recombinant I (Roche, 04716728001) according to manufacturer’s instructions. RNA was quantified using Nanodrop 1000 (ThermoFisher Scientific, Waltham, MA, USA), and 1 µg of RNA was transcribed using the Affinity Script Multi Temp RT kit (Agilent 600107-51) according to manufacturer’s instructions.

### 2.8. RT-PCR

Samples were assayed in triplicate using the SYBR Green I Master Mix (Roche, 04707516001), 0.25 µM of gene specific forward and reverse primers, and 25 ng of cDNA, adjusted to a final volume of 20 µL. RT-PCR was performed on a LightCycler 480 II instrument (Roche, Basel, Switzerland). RT-PCR program: 10 min 95 °C, 45 cycles of: 95 °C 30 s, 20 s at 60 °C and 72 °C for 20 s. Dissociation curve analysis was performed at the end of each program. One sample of the amplicon for each gene was run on 1% agarose gel to verify the specificity of the primers. Primer pairs with annealing temperatures and extension times are provided in Table S2.

Transcript levels were normalized relative to β-actin. Relative expression was determined using the method described by Pfaffl [21] Three independent replicates were carried out for each gene at each time point. Relative gene expression, standard error and significance were determined using a *t*-test on the dCT values for each biological sample, *p* < 0.05 was deemed as significant.

### 2.9. RNA Sequencing Library Preparation, Sequencing and Bioinformatics Analysis

Extracted RNA quality was assessed on a Fragment Analyzer (Advanced Analytical Technologies, Inc., Ankeny, IA, USA). RNA-seq libraries were prepared using the Illumina TruSeq Stranded mRNA reagents (Illumina; San Diego, CA, USA), according to manufacturer’s instructions with no deviations to the standard protocol, with the starting amount of 1 μg total RNA. Cluster generation was performed with the resulting libraries using the Illumina TruSeq SR Cluster Kit v3 reagents and sequenced on the Illumina HiSeq™2000 using TruSeq SBS Kit v3 reagents by Lausanne Genomic Technologies Facility (GTF) at the Centre for Integrative Genomics at University of Lausanne (UNIL), Lausanne, Switzerland. Sequencing data were processed using the Illumina Pipeline Software version 1.82 by Dr Sylvain Pradervand and Dr Sandra Calderon of GTF at UNIL. Quality control steps on the raw fastq files included: per base sequence quality, per sequence quality scores, per base sequence content, GC content (per base and sequence) and sequence duplication levels. Purity-filtered reads were adapter and quality trimmed with Cutadapt [22] (v. 1.2.1). Remaining reads were further filtered for low complexity with PrinSeq (v. 0.20.3). Reads were aligned against Danio rerio Zv9 genome using Tophat aligner (v. 2.0.9, with bowtie v. 0.12.9). The number of read counts per gene locus was summarized with htseq-count [23] (v. 0.5.4p3) using *Danio rerio* Ensembl 73 gene annotation. Quality of the RNA-seq data alignment was assessed using RSeQC [24] (v. 2.3.7). Statistical analysis was performed for genes and isoforms with the Bioconductor package DESeq (R v. 1.12.1) for normalization and limma (R v. 3.12.3) for differential expression. The steps carried out for the analysis included the DESeq normalization, VST, linear model for differential expression of the 4 groups and extraction of contrasts using the control condition as a reference. Adjusted *p*-values were computed for each comparison by the Benjamini–Hochberg method, controlling for FDR with cut off at 0.05. RNA sequencing analysis based on 12,088 probes identified 2625 differentially expressed genes (Table S3). Additionally, genome-wide significant threshold (0.05/12088 = 4.13 × 10^−6^) at all time points identified 66 differentially expressed genes.

Data distributions were inspected globally, and R base *scale* v1.0.4 method was used to center and scale fold-change expression per-gene across three time points. Genes were clustered based on their expression pattern. The *dist* function of Stats v3.6.2 by R base was used for distance matrix computation to compute the distances between the rows of the expression fold-change data matrix (row, gene ID; column, timepoint). The *hclust* function from the same package was used for hierarchical cluster analysis and *cutree* for determining clusters based on tree height. *Heatmap2*, from gplots, was used for illustration of heatmap and dendrogram. Cluster IDs are listed in Table S4.

Gene expression cluster groups were tested by pathway analysis based on their functional annotation. DAVID Bioinformatics Resources 6.8 NIAID/NIH (organism *Danio rerio*) was used for querying each of the 13 expression clusters via ENSEMBL gene ID list. Novel genes lacking a matching ENSEMBL ID in DAVID were excluded. Annotation parameters consisted of: functional categories, gene ontology, general annotations, literature, main accessions, pathways, and protein domains. Functional annotation clustering was used to prioritize enriched pathways. Default analysis options were used; however, classification stringency was set to *high* to reduce false positives. Bonferroni corrected *p*-values were used for strict multiple test correction. A summary of query and results are listed in Table S4.

The top enriched pathways were assessed using STRING db v11.0 via multiple query list with organism *Danio rerio.* The parameters *physical network evidence* (edges) and highest confidence 0.9 were used to prioritize closely related proteins.

## 3. Results

### 3.1. Progression of the Induced Lesion with Cryoinjury

**Figure 1:**
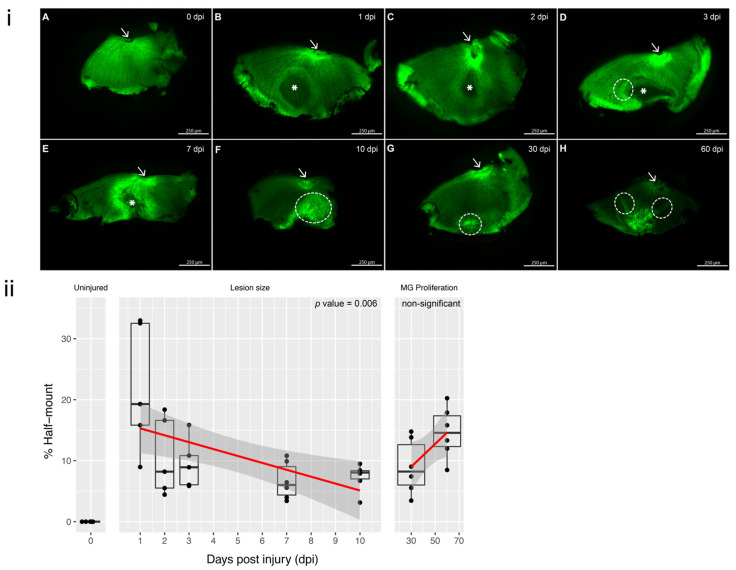
GFP retinal half-mounts showing the resolve of the lesion with the remaining scar; arrows point to the optic nerve for easier orientation: (**i**) (**A**) Uninjured (0 dpi) retinal half-mount with no lesion. (**B**,**C**) 1 and 2 dpi: lesion area is represented with a *, corresponding to a darker shaded area within the half-mount. (**D**) 3 dpi: the original circular lesion (*) has changed and there is a brighter margin surrounding the left side of the lesion (circled). (**E**) 7 dpi: reduced size of the lesion (*) and increased fluorescence within the margin. (**F**) 10 dpi: closing of the lesion with increased intensity of fluorescence, CMZ has been disrupted (circled). (**G**) 30 dpi: the lesion has resolved, and only reduced margin is visible near the CMZ (circled). (**H**) 60 dpi: the margin shows gathering of the tissue as the retina reaches the defect area, resulting in a fold at the site (circled). Each half-mount represents a separate animal. Scale bars represent 250 µm. (**ii**) Surrounding MG fluorescence intensity. The response to cryo-injury was quantified by calculating the percentages of the lesion area relative to the total area of the retinal half-mount. Linear regression was performed for (1) lesion size throughout 1–7 dpi and (2) MG proliferation between 10 and 60 dpi. A strong association for lesion size reducing from 1 to 10 dpi (*p* value = 0.00626). MG proliferation was notably increased from 10 dpi (*p*-value 0.0508, ns). Time points 1 dpi, 2 dpi and 3 dpi n = 5 independent animals and time points uninjured, 7 dpi, 10 dpi, 30 dpi and 60 dpi contain n = 6 independent animals.

In order to accurately assess the lesion size and the extent of residual defect, we imaged retinal ‘half-mounts’ of Tg(*gfap:GFP*)mi2001 zebrafish (ZIRC, Eugene, OR), which express green fluorescent protein (GFP) in glial cells under a glial fibrillary acidic protein (GFAP) promoter, at time points from 1-day-post injury (dpi) to 60 dpi in comparison to uninjured control (Figure 1A). Retinal ‘half mounts’ were used as opposed to retinal ‘full mounts’ due to the small size of the zebrafish retina and its weak ability to detach in single mount. At 1 and 2 dpi, a clear ‘dead area’ was easily visualized at the lesion site (Figure 1B,C), which corresponded to 20.45% of the total half-mount area at 1 dpi and 10.24% at 2 dpi. At 3 dpi, the lesion represented 9.09% of the total area of the retinal half-mount and displayed a notable margin surrounding the lesion area (Figure 1D, circle). The size of the lesion continued to reduce at 7 dpi with the lesion representing 6.7% of the total half-mount (Figure 1E). At 10 dpi, the lesion was no longer visible as ‘dark area’ but instead the margin had become enlarged and covered the lesion site, presenting as enlarged margin fluorescent area in comparison to the intact retina, representing 10.21% of the total area of half-mount (Figure 1F). These are most likely cells that have survived in the lesion area and have begun re-expressing GFAP; however, it is possible the fluorescence came from cells that have migrated into the lesion area from the periphery and initiated the dedifferentiation and proliferation of MG, thus beginning the regeneration process. At 30 dpi, the proliferation of GFAP + ve cells continued, and the area of proliferation equated to 8.17% of the total half-mount area (Figure 1G). From the regeneration of the lesion, it was evident that the dead cells were cleared, apparent by the absence of dark area at 30 dpi, indicating that the defect seen at later dpi was not an abscess of dead cells that persisted. Through 60 dpi, the proliferation corresponded to 14.73% of the total half-mount area, clearly signifying the increased proliferation of dedifferentiated MG (Figure 1H). At 60 dpi, there was evidence that, in some cases, the eye had become ‘gathered’ at the site of the defect. This could indicate that the repair of the lesion was linked to a closure of the surrounding surviving tissue and excision of the dead area, which can be seen by folds extending from the defect area at 60 dpi (Figure 1H). It is important to note that the overlap between reducing lesion area and the proliferation of dedifferentiated MG made the detection of accurate, early proliferation difficult. Additionally, although the initial lesion was always performed caudal to the ciliary marginal zone (CMZ), in many cases during the regeneration, the lesion area seemed to have migrated and disrupted the CMZ.

### 3.2. Partial Regeneration Observed by Histology

**Figure 2:**
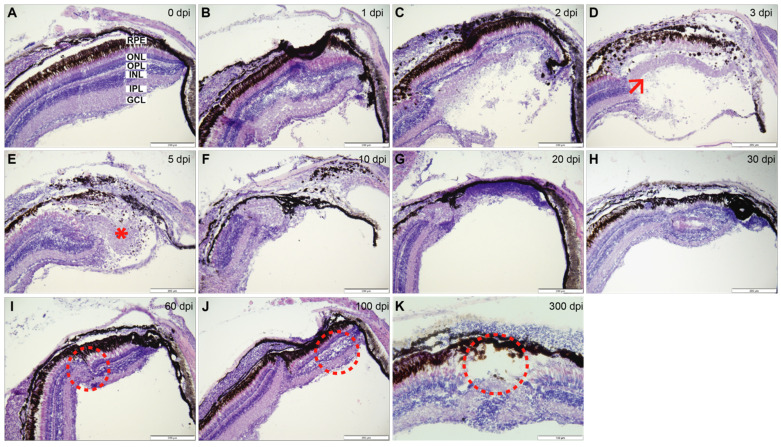
Hematoxylin and eosin staining: (**A**) Control staining in uninjured retina (0 dpi) with distinct retinal layers. (**B**) 1 dpi: edema in the lesion area particularly affecting the IPL and INL. (**C**) 2 dpi: severe edema causing a major disruption to majority of the retinal layers. (**D**) 3 dpi: degeneration phase, coagulative necrosis involving all retinal layers and the formed ‘blister’. Low numbers of polymorphonuclear and mononuclear inflammatory cells were detected (arrow). (**E**) 5 dpi: remodeling phase, ‘blister’ is still present, expansion of and piling of retinal cells at the lesion periphery coincide with maximal proliferation levels (*). (**F**) 10 dpi: lesion size noticeably smaller in comparison to previous time points. (**G**) 20 dpi: lesion size reaches the thinnest point, clearing of dead cells complete. (**H**) 30 dpi: regrowth phase, largely regenerated retina, a clearly dysplastic region is evident exhibiting a mixture of retinal cells and absence of lamination. (**I**,**J**) 60 and 100 dpi: focal retinal reorganization, reduced number of rods and cones, inward growth of RPE, focal disruption and convergence of INL and ONL (circled). (**K**) 300 dpi: incomplete regeneration presenting the absence of photoreceptor outer segments (circled), dysplasia apparent with largely normal retina surrounding the affected area. Retinal layers labelled in control panel for reference; RPE—retinal pigment epithelium, ONL—outer nuclear layer, OPL—outer plexiform layer, INL—inner nuclear layer, IPL—inner plexiform layer, GCL—ganglion cell layer. 300 dpi at 40× with scale bar representing 100 µm to show the full regeneration process. Rest of scale bars represent 200 µm.

**Figure 3:**
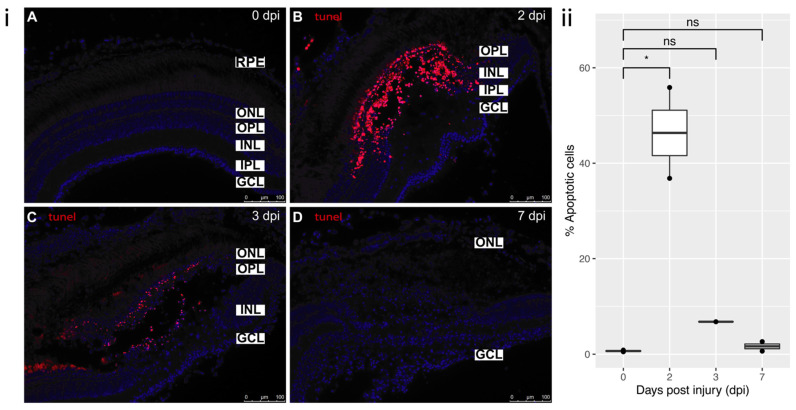
TUNEL staining: (**i**) (**A**) Control staining in uninjured retina (0 dpi). (**B**) 2 dpi: profound localized cell death in the area of cryoinjury, degeneration phase. Predominantly affected layers were OPL and INL. (**C**) 3 dpi: cell death still ongoing, but to a lesser extent than at 2 dpi, clearing off the dead cells by macrophages. (**D**) 7 dpi: damaged nuclei are no longer detected in the retina, reprogramming of MG, remodeling phase. All sections were counterstained with DAPI. Apoptotic cells—TMR Red; Nuclei, DAPI—blue. Retinal layers labelled in control panel for reference; RPE—retinal pigment epithelium, ONL—outer nuclear layer, OPL—outer plexiform layer, INL—inner nuclear layer, IPL—inner plexiform layer, GCL—ganglion cell layer. Scale bars represent 100 µm. (**ii**) Relative percentage of apoptotic cells at each time-point (n = 2), * *p* value = 0.02.

At early time points, 1 through 3 dpi (Figure 2B–D), focal coagulation necrosis consistent with cryoinjury was present, showing the highest rate of cell death (Figure 3). Within the first 3 dpi, a blister formed with low numbers of polymorphonuclear and mononuclear inflammatory cells (Figure 2D). In some cases, retinal detachment was evident (data not shown).

**Figure 4:**
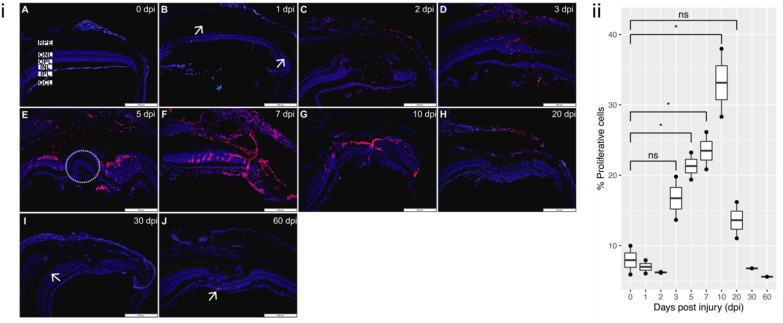
PCNA staining of proliferation: (**i**) (**A**) Proliferation is minimal in the control uninjured retina (0 dpi). (**B**) 1 dpi: proliferation begins to be detected (arrows). (**C**,**D**) 2 dpi and 3 dpi: proliferation more evident at the lesion site. (**E**) 5 dpi: proliferation pronounced, with positive nuclei located predominantly in the ONL at the lesion margin, absent in the lesion site (circled). (**F**) 7 dpi: proliferation now detected throughout the lesion. (**G**,**H**) 10 dpi and 20 dpi: proliferation started to decrease as new cells have started to repopulate the lesion site. (**I**,**J**) 30 dpi and 60 dpi: proliferation now only marginally detected (arrows). All sections were counterstained with DAPI. PCNA proliferation marker—red; Nuclei, DAPI—blue. Retinal layers labelled in control panel for reference; RPE—retinal pigment epithelium, ONL—outer nuclear layer, OPL—outer plexiform layer, INL—inner nuclear layer, IPL—inner plexiform layer, GCL—ganglion cell layer. Scale bars represent 200 µm. (**ii**) Relative percentage of proliferative cells at each time-point (n = 2), * *p* value < 0.05.

During the regeneration phase, 5 through 30 dpi (Figure 2E–H), proliferation of cells located in INL and ONL was noted. Immunohistochemistry staining revealed that these cells were proliferating cell nuclear antigen (PCNA) positive and displayed a characteristic notched nucleus that was associated with MG cell processes (Figure 4). The injured retina appeared most similar to the uninjured retina at 30 dpi; although, even at this time, point dysplasia was evident (Figure 2H). Most notably, there was often a failure to recreate retinal lamination. In the later time points, 60, 100 and 300 dpi, focal areas of dysplasia persisted (Figure 2I–K). A key hallmark was the absence of photoreceptor outer segments (Figure 2K). No evidence of fibrosis was noted during retinal regeneration.

### 3.3. Cell Death and Proliferation

#### 3.3.1. Cell Death

Cell death occurred rapidly following cryoinjury, peaking at 2 dpi, with damaged nuclei removed by 7 dpi (Figure 3B,D, respectively). Cell death is particularly seen to be restricted to INL and OPL layers. TUNEL is known to label not only apoptotic cells, but also early necrotic cells [25]. These results agree with the half-mount and histological data (Figure 1 and Figure 2) showing that the cells die through coagulative necrosis with ‘ghosting’ of cells present at 2 dpi, and that the debris is cleared after this time point, resulting in almost no detectable TUNEL staining at 7 dpi.

#### 3.3.2. Proliferation

Control retina and 1 dpi retina were minimally mitotically active (Figure 4A,B). At 2 and 3 dpi, there was a pronounced proliferation occurring at the lesion margin (Figure 4C,D) and PCNA positive cells were present in all retinal layers and RPE. Proliferation was highest at 5 and 7 dpi (Figure 4E,F). PCNA positive cells were initially concentrated in the ONL at the lesion margin but at later time points migrated to the lesion site. By 30 dpi all through 60 dpi, proliferation rate has dramatically decreased (Figure 4I,J).

The duration of cell death and clearing, as well as proliferation, are consistent with previously published zebrafish retinal injury models [16,17,18]. Despite the fact that cryoinjury results in persistent dysplasia, we do not note any substantive alterations in quantity, duration or the cell type involved in cell death and proliferation in this particular injury model.

### 3.4. Cellular Components of Residual Defect

**Figure 5:**
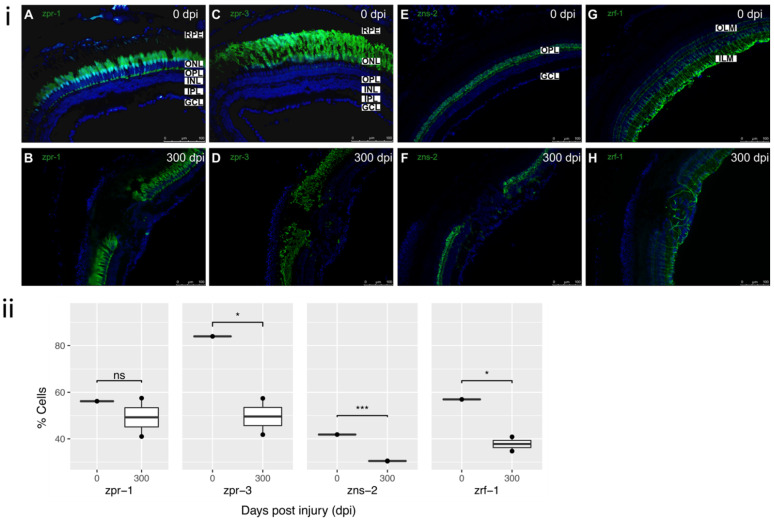
Cellular components of defect at 300 dpi: (**i**) (**A**) Uninjured retina (0 dpi) showing typical staining of cones with zpr-1 antibody. (**B**) Absence of restoration of cone photoreceptors within the injured retina at 300 dpi. (**C**) Uninjured retina (0 dpi) showing typical staining of rods with zpr-3 antibody. (**D**) Incomplete restoration and disorganization of rod photoreceptors within the lesion at 300 dpi. (**E**) Uninjured retina (0 dpi) showing typical staining of horizontal cells with zns-2 antibody. (**F**) Absence of horizontal cells within the lesion at 300 dpi. (**G**) Uninjured retina (0 dpi) showing typical staining of MG with zrf-1 antibody. ILM and OLM labelled for reference. (**H**) Partial restoration of MG within the residual lesion at 300 dpi. All sections were counterstained with DAPI. zpr-1, zpr-3, zns-2, zrf-1 markers—green; Nuclei, DAPI—blue. Retinal layers labelled in control panel A for reference; RPE—retinal pigment epithelium, OLM—outer limiting membrane; ONL—outer nuclear layer, OPL—outer plexiform layer, INL—inner nuclear layer, IPL—inner plexiform layer, GCL—ganglion cell layer; ILM—inner limiting membrane. Images (**A**,**C**) at 40×, rest of images at 20×. Scale bars represent 100 µm. (**ii**) Relative percentage of cell markers (zpr-1, zpr-3, zns-2, zrf-1) at 0–300 dpi (n = 2), * *p* value < 0.05; *** *p* value <0.005.

The presence of a dysplastic region at 300 dpi was an unexpected finding since zebrafish is capable of regenerating an intact retina after injury [10,16] (Figure 2). Due to this finding, the cellular components; red-green cones, rods, horizontal cells and MG, of the defect were investigated at 300 dpi (Figure 5).

#### 3.4.1. Red-Green Double Cones

During zebrafish development, red and green cones are the first cone opsins to be expressed, eventually forming the fused red-green double cones. These photoreceptors are arranged in parallel lines within the normal retina [26,27]. The typical staining pattern for an intact retina was evident in the control tissue (Figure 5A). Following the injury, cones have not restored within the lesion in the majority of the samples tested, and the cells did not exhibit an expression pattern suggesting an intact and functioning retina (Figure 5B). The presence of double cones in the dysplastic region varied between samples, being completely absent in some cases, while representing some portion of the defect in others (data not shown).

#### 3.4.2. Rods

Rod photoreceptors are the first to differentiate during development and the first to reappear following injury, which may be due in part to the continuous production of rod photoreceptors throughout the zebrafish life span [28]. Uninjured retinas showed typical staining for rod outer segments (Figure 5C). In the injured retinas, disorganized rods were present in the lesion margin, but not within the dysplastic area (Figure 5D).

#### 3.4.3. Horizontal Cells

In uninjured zebrafish retina, zns-2 labelled cells in the ONL, and weakly stained horizontal cells and fibers in the OPL (Figure 5E). In 300 dpi retina, zns-2 staining was weekly visible throughout the center of the lesion; however, it did not display any recognizable pattern (Figure 5F).

#### 3.4.4. Müller Glia (MG)

In the intact retina MG bodies reside in the INL and send processes that span the thickness of the retina to form the outer and inner limiting membrane (OLM and ILM) (Figure 5G) [4,29]. Examining the residual dysplastic area at 300 dpi, MG were present with processes extending irregularly throughout the defect and the well-ordered vitread–sclerad orientation was lost (Figure 5H).

The morphology of MG and the aberrant occurrence of other retinal cell types within the residual defect are consistent with a mammalian state of reactive gliosis [29]. The cryoinjury method reliably produced this persistent reactive gliosis in the majority of induced lesions. Reactive gliosis in zebrafish is well-known in the early phases of retinal regeneration and resolves as the retina repairs [30]. What makes the results of this cryoinjury unique is the persistence of gliosis and the failure of the retina to fully repair, even throughout the timeline of 300 dpi (Figure 2 and Figure 5).

### 3.5. Modulation of Transcript Levels during the Degeneration, Proliferation and Regeneration Processes

We performed RT-PCR to examine signaling during the degeneration, proliferation and regeneration phases following cryoinjury in comparison to uninjured retina (0 dpi). RT-PCR was directed at the following pathways previously reported to play a role in retinal regeneration: achaete–scute complex, a developmental complex often activated in neural tissue regeneration [31]; *stat3* required for maximum MG proliferation [32]; extracellular matrix (ECM) components (*mmp2*, *mmp9* and *tgfbi*) demonstrated as conducive to retinal regeneration in a mouse model [28,33]; and *cxcr5* and *gata3*, two genes that have been shown to drive neural progenitor cell proliferation in the telencephalon of zebrafish, but have not yet been investigated in retinal regeneration [34,35].

**Figure 6:**
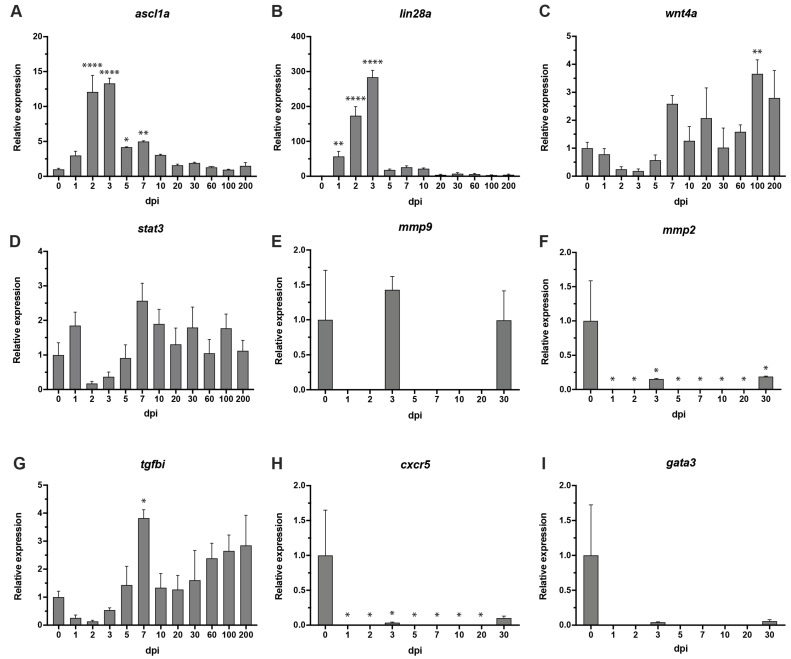
RT-PCR: (**A**) *ascl1a* was significantly expressed in the early stages of regeneration beginning at 2 dpi through to 7 dpi. (**B**) Significant expression of *lin28a* from 1 to 7 dpi. (**C**) *wnt4a* showed significant upregulation at 100 dpi. (**D**) *stat3* showed no differential expression throughout any of the time points, 1–200 dpi. (**E**,**F**) *mmp9* showed no changes in expression, *mmp2* was downregulated throughout all time points. No expression was detected at time points 1 dpi, 2 dpi, 5 dpi, 7 dpi, 10 dpi and 20 dpi for both, *mmp9* and *mmp2*. (**G**) *tgfbi* showed significant change in expression at 7 dpi. (**H**) *cxcr5* showed differential expression at 1–30 dpi. No expression was detected at time points 1 dpi, 2 dpi, 5 dpi, 7 dpi, 10 dpi and 20 dpi. (**I**) *gata3* reported no significant differential expression at any of the time points. No expression was detected at time points 1 dpi, 2 dpi, 5 dpi, 7 dpi, 10 dpi and 20 dpi. Uninjured retina—0 dpi. β-actin served as reference gene. Three independent replicates were carried out for each gene at each time point. Error bars represent +/− standard error of the mean (SEM). Stars used to denote significance * *p* value < 0.01, ** *p* value < 0.005, **** *p* value < 0.0001.

Consistent with other zebrafish retinal injury paradigms, increased achaete–scute homolog 1 expression was observed [10,11] starting at 2 dpi (12-fold expression, *p* < 0.0001). *ascl1a* expression peaked at 3 dpi (13-fold increased expression, *p* < 0.0001) and remained significantly elevated through 5 dpi and 7 dpi (4-fold and 5-fold upregulation, *p* < 0.05 and *p* < 0.01, respectively) (Figure 6A). Following cryoinjury, substantive alterations in lin-28 homolog A (*lin28a*) expression were observed as previously reported [11]. Significant upregulation of *lin28a* started at 1 dpi (69-fold, *p* < 0.01), 2 dpi (210-fold, *p* < 0.0001) and peaked at 3 dpi (345-fold, *p* < 0.0001) over basal levels of the uninjured control at 0 dpi. *lin28a* expression then began declining gradually until 200 dpi (Figure 6B). Wingless-type MMTV integration site family, member 4 (*wnt4a*), is regulated by Ascl1a through the Lin-28 independent pathway, was upregulated from 7 dpi with significant upregulation at 100 dpi (3.8-fold, *p* < 0.01), which coincides with previously published data [36] (Figure 6C).

There was no differential expression noted for *stat3* for any of the time points, contrary to previous report showing expression of *stat3* as early as 5 to 36 h of constant light treatment to the zebrafish retina [32] (Figure 6D).

No significant up- or downregulation was observed for matrix metalloproteinase-9 (*mmp9*). Differential expression was noted for matrix metalloproteinase-2 (*mmp2*) at all time points, downregulation was observed from 1 through to 30 dpi (*p* < 0.05 for all time points) (Figure 6E,F). Significant upregulation in expression was noted in transforming growth factor, TGF-β induced (*tgfbi*) at 7 dpi (4-fold, *p* < 0.05). No other time points noted any differential expression (Figure 6G).

Significant downregulation was noted at all time points (1–20 dpi, *p* < 0.05) in comparison to uninjured control at 0 dpi (Figure 6H). These data partly contradict previously published studies on *cxcr5* in zebrafish brain, which showed the upregulated expression until 3 dpi before returning back to basal levels at 7 and 15 dpi [35].

No significant up- or downregulation was noted at any of the time points for *gata3* (Figure 6I). These data are in contradiction with previously published data that show upregulation of *gata3* from 1 through to 7 dpi. The expression was no longer detected at 14 dpi, which is in line with the data presented here [34].

### 3.6. RNA Sequencing Analysis

**Figure 7:**
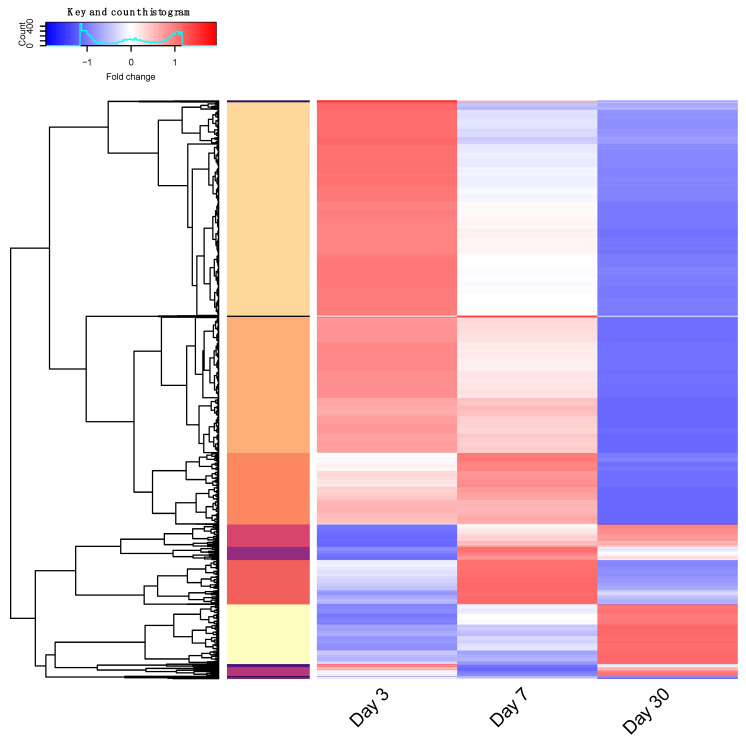
RNAseq analysis gene clustering. Genes showing differential expression over three time points scaled per-gene. Hierarchical clustering was performed, and thirteen distinct groups determined by tree height are illustrated by color in the left vertical bar. Fold change in gene expression is represented by color over days 3, 7, and 30; low—blue to high—red. Scaled expression data are listed in Table S3. Genes with common expression profiles were assessed for protein pathway enrichment. Query summary and significant associations listed in Table S4.

RNA sequencing (RNA-seq) analysis was carried out to identify differentially expressed genes. DESeq normalization and variance stabilization transformation (VST) was performed, followed by creating a linear model for differential expression of four time point groups (0 dpi, 3 dpi, 7 dpi and 30 dpi). Comparison was made against the control condition (0 dpi) as reference. Up- and downregulated genes with a false discovery rate (FDR) < 0.05 and fold change (FC) > 2 were extracted for further analysis (Table 1, Figure S2). The scaled FC for differentially expressed genes is illustrated in Figure 7 and Table S3. Hierarchical clustering was performed to cluster genes with shared expression profiles. Each gene cluster was assessed by functional annotation with DAVID v6.8. After correction for multiple testing, nine significantly enriched protein pathways were identified from five DEG clusters, eight functional annotation clusters (Table 2 and Table S4). Briefly, the enriched pathways included: ribosome, eukaryotic translation initiation complex, TCP-1, Kinesin, Op18/stathmin, collagen and MHC class II proteins. Genome-wide significance at all three time points showed 66 differentially expressed genes, which were associated with seven protein pathways (Figure S1 and Table S4 sheet ‘FDRgenomeSignif’).

## 4. Discussion

Cryoinjury to the retina seems to induce an incomplete retinal regeneration, markedly less successful than other injury paradigms for the zebrafish [9,10,11,12,13,14,15,16,17,18]. This method of injury has been previously used to study degeneration in zebrafish cardiac ventricle, which described a transient scar with degradation of the scar tissue [35], myocardium and caudal fin [37,38]. The initial response (from 1 to 20 dpi) in the model described here, is similar to the changes and timing of responses of other retinal injury models [18,34,35,39,40,41]. The innate immunity response within the retina consists of microglial cells and MG [42,43], which act via phagocytosis to eliminate the dead cell bodies within the damaged retina [44]. This clearing process then stimulates regeneration [45] and the phagocytic activity of MG decreases once the progenitors proliferate and repopulate the lesion site [11,46,47,48]. This process can be seen in our model to a limited extent as we show convergence of the INL and ONL and evidence of dividing MG at the lesion site. However, the regeneration was incomplete, resulting in the disorganization of retinal layers. Equally, it was previously described that the progenitors are able to regenerate most of the cell types in injury damaged retina [9,11,49,50]. We have found this was not the case when cryoinjury was used as method of injury. None of the cell types analyzed in this study, apart from disorganized MG, were able to fully repopulate and regenerate the lesion site.

The cryoinjury model evidently diverges from other injury models at around 30 dpi, as it never fully recovers from the initial injury, leaving a dysplastic region characterized by incorrect or incomplete lamination, and a focal absence of photoreceptors in the outermost layer. Even though it is well established that zebrafish possess the ability to regenerate retina following injury [49,51], other data demonstrate that the inhibition of MG proliferation results in reactive gliosis [52]. While unsuccessful regeneration has previously been induced by using transgenic fish to knock out expression of critical genes in the regeneration pathway [3,32,39,51,53,54,55], no reports of WT zebrafish sustaining persistent gliosis are found in the literature. As such, we feel our cryoinjury model, although “less successful” in regeneration, may offer unique insights into gliotic versus neuro-regenerative responses, and the pathways and mechanisms involved in shifting the balance from neurogenic to gliotic and vice versa.

Only recently has doubt been cast on the concept of perfect healing in the zebrafish. A recent study showed a regenerated heart that exhibited anomalies that extended through 180 dpi. The authors suggested that these changes could be permanent, and that differences in initial injury severity might influence the outcome [56]. This is consistent with our model of cryoinjury. In our study, we tested a 5 s retinal cryoinjury against 1 s application with the same experimental set up as in Hein et al. [56]. Initially, the 5 s cryoinjury produced a larger lesion, accompanied by increased cell death, but ultimately resolved to be indistinguishable from the 1 s injury (data not shown). More research needs to be conducted to explain these differences and the factors that may play a role in this scenario.

Gene expression analysis during the recovery period showed expression consistent with previously established retinal regeneration pathways, particularly *ascl1a*-dependent *lin28a* expression [11]. Ascl1a, a marker of proliferating MG, is directly involved in MG reprogramming, regulated by Jak/Stat signaling [57], and has previously been shown to be necessary for zebrafish retinal regeneration [11,51]. In our data, the upregulation of *ascl1a* was noted as early as 2 dpi through to 7 dpi. Equally, the expression of *lin28a*, necessary for MG reprogramming and subsequent proliferation in injured zebrafish retina, was detected early in the regeneration process at 1–3 dpi [32,55]. Expression of *wnt4a*, regulated by Ascl1a through the Lin-28 independent pathway, was detected at all time points with significant upregulation at 100 dpi. *stat3* was first shown to be upregulated in light-damaged retina, and is essential for maximum MG proliferation [32,58,59]. Expression of *stat3* post light injury was noted as early as 5 h [32]; however, in our injury model there was no significant upregulation of *stat3* at any of the time points tested. These data signify that the regeneration process induced via cryoinjury was predominantly activated as previously documented [10,11,36].

Cxcr5 was previously identified as playing a permissive role in dividing radial glial cells, and the specification of neuronal cell fate in the zebrafish telencephalon [35]. Likewise, the transcription factor *gata3* has previously been reported as necessary for regeneration in the zebrafish telencephalon, heart and fin [34]. We were interested to examine if either of these genes play a significant role in the regenerating retina. Expression levels of *cxcr5* and *gata3* did not follow the patterns observed in the regenerating telencephalon of zebrafish; therefore, it may be that neural regeneration within the brain and retina are regulated by different or divergent pathways [2,34,39].

The ECM plays important, and contradictory, roles in tissue regeneration. Fibronectin and collagen act as both a scaffold for tissue synthesis, and an impediment to cell invasion [60]. The gelatinases, Mmp2 and Mmp9, have been demonstrated as conducive to retinal regeneration in a mouse model [28]. Both gelatinases have also been shown to play a role in zebrafish fin and newt limb regeneration [26,61]. Tgfbi is a component of the ECM, upregulated in zebrafish fin regeneration, downstream target of TGF-β (downregulator) and Mmp9 activator and serves as a pro-apoptotic protein in the retina [27,62,63,64]. Therefore, we were interested to see if *mmp2*, *mmp9* and *tgfbi* were differentially expressed following cryoinjury. Significant downregulation of *mmp2* was observed from 1 to 30 dpi; however, no significant differential expression was noted for *mmp9*, which contradicts previously published data in zebrafish and in mice [26,28]. Significant upregulation of *tgfbi* was noted at 7 dpi, previously shown to be highly expressed in the regeneration of zebrafish fin at 5 dpi [27]. Additionally, there were no significant changes in the expression of several genes related to the TGF-β-signaling pathway essential for tissue repair [27,65]: *tgfbr1* and *tgfbr2* and *tgfb1a*, *tgfb2* and *tgfb3* (data not shown).

Our model recapitulates several of the key features of regeneration seen in other models. However, several of the previously reported genes did not have significantly different expression in our model. These genes may be responsible for incomplete regeneration but cannot be verified until future studies of incomplete regeneration are reported. Previously reported genes and pathways of regenerative response include the asymmetric cell division of MG cells to produce neuronal progenitor cells, β-catenin/Wnt, TGF-β signaling, alpha-1 tubulin, Pax6, Crx, N-cadherin, microglia and others as reviewed by Stella et al. [66] and Gao et al. [67].

Analysis of gene expression by RNA sequencing with 12,088 probes identified 2625 significant differentially expressed genes with an in vivo injury model. Additionally, 66 genes had a genome-wide significant change in expression for all time points, associating with seven known significantly enriched protein pathways. These candidate genes both, with and without well-defined biological functions, may offer further insight; showing a strong change in expression as a monogenic response to damage or as part of a larger protein pathway.

Although this model does result in a non-resolving portion of the lesion, there was no histological evidence of fibrosis in the injury area during regeneration or in the final residual defect noted in our model. Thus, the defect appears to be a disorganized collection of retinal cells, predominantly MG exhibiting thickened GFAP+ processes, but also sometimes including cone and/or rod photoreceptors. These features are also the hallmarks of a gliotic scar in mammals [6].

One caveat of the cryoinjury model is the comorbidity of lesion with retinal detachment. Neural cells, particularly of the retina, tend to pile up and form rosettes when they are dislodged from their normal orientation, and any detached retina will be a candidate for abnormal clumping and rosette formation [68]. Regeneration is a process requiring various direct cellular and/or extracellular matrix interactions, which may be missing or reduced when the retina is detached [69,70]. We suspect that it will prove impossible to perform cryoinjury to the retina without frequently also inducing retinal detachment, preventing discernment of the effects, and determining which effects are due to subsequent retinal detachment. There do not seem to exist any published models of retinal detachment in the zebrafish for comparison.

Another aspect that needs further investigation is the age of the zebrafish and whether it could play a role in the incomplete regeneration of the retina. In other published studies, zebrafish were seen to have fully regenerated their retinas at 105 dpi and 63 dpi [10,15]. Therefore, we do not suspect that the age of the zebrafish at these time points would have a direct effect on regeneration; however, we cannot exclude the possibility of age having an impact after 100 dpi.

We further hypothesize that cryoinjury may be causing irreparable cell damage to the retina due to the hindering of the appropriate stimulus, which does not induce complete dedifferentiation of MG into progenitors. Of critical importance is the understanding of the pathways and mechanisms underlying successful and, imperatively, unsuccessful regeneration in order to recreate the regeneration process for clinical applications.

## 5. Conclusions

We demonstrate that the regeneration of the zebrafish retina does not always occur as previously reported, and complete regeneration is dependent on the type of injury used. To our best knowledge, cryoinjury to the retina has not previously been used as an injury model in zebrafish. By introducing this simple type of injury, which gives a result comparable to the mammalian failure to regenerate, we believe that this model proves to be useful in comparative and translational studies of regeneration and in modelling human diseases.

## Figures and Tables

**Table 1:** Total number of genes and isoforms with significant differential expression. Three experimental conditions (3 dpi, 7 dpi and 30 dpi) are specified in comparison to control (0 dpi). FDR cut off <0.05. dpi = days post injury.

**GENES**						
Comparison	FDR < 0.05			FDR < 0.05 and fold change >2		
	ALL	UP	DOWN	ALL	UP	DOWN
3 dpi_vs_0 dpi	12088	6507	5581	6014	4157	1857
7 dpi_vs_0 dpi	10142	5625	4517	4446	3682	764
30 dpi_vs_0 dpi	2939	2489	450	1500	1481	19
**ISOFORMS**						
Comparison	FDR < 0.05			FDR < 0.05 and fold change >2		
	ALL	UP	DOWN	ALL	UP	DOWN
3 dpi_vs_0 dpi	13362	7979	5383	7655	5645	2010
7 dpi_vs_0 dpi	10228	6343	3885	5454	4562	892
30 dpi_vs_0 dpi	2476	2205	271	1673	1529	144

**Table 2:** Functional pathway annotation of DEG. Differentially expressing genes were clustered into distinct expression profile groups (Figure 7, and extended Table S4). Gene lists from each group were used to query DAVID v6.8 as candidates of shared protein pathways. Significantly enriched pathways were identified using string selection criteria and Bonferroni correction for multiple testing. A full list of gene names is shown in Table S4. * DEG cluster number based on expression profile (as shown in Figure 7). ** DAVID functional annotation pathway cluster. *** Bonferroni corrected *p* value.

	DEG Cluster *	Annotation Cluster **	Enrichment Score	Category	Term	Gene Count	% of Pathway	Fold Enrichment	*p* Value ***
All significant DEG	2	1	12.09	GOTERM_CC_DIRECT	GO:0005840~ribosome	34	4.21	5.59	2.71 × 10^−13^
	2	1	12.09	UP_KEYWORDS	Ribosomal protein	26	3.22	6.09	1.51 × 10^−10^
	2	1	12.09	KEGG_PATHWAY	dre03010:Ribosome	31	3.84	4.25	1.34 × 10^−09^
	2	1	12.09	GOTERM_MF_DIRECT	GO:0003735~structural constituent of ribosome	31	3.84	4.21	3.49 × 10^−08^
	2	2	4.44	INTERPRO	IPR002194:Chaperonin TCP-1, conserved site	6	0.74	21.61	3.39 × 10^−3^
	2	2	4.44	INTERPRO	IPR027410:TCP-1-like chaperonin intermediate domain	6	0.74	19.21	7.39 × 10^−3^
	2	2	4.44	INTERPRO	IPR017998:Chaperone tailless complex polypeptide 1 (TCP-1)	6	0.74	19.21	7.39 × 10^−3^
	2	2	4.44	INTERPRO	IPR027409:GroEL-like apical domain	6	0.74	14.41	4.18 × 10^−2^
	2	2	4.44	INTERPRO	IPR027413:GroEL-like equatorial domain	6	0.74	14.41	4.18 × 10^−2^
	2	3	3.55	INTERPRO	IPR019821:Kinesin, motor region, conserved site	10	1.24	6.26	3.16 × 10^−2^
	2	3	3.55	SMART	SM00129:KISc	10	1.24	5.96	1.00 × 10^−2^
	3	1	26.82	GOTERM_CC_DIRECT	GO:0005840~ribosome	42	8.55	11.27	2.72 × 10^−29^
	3	1	26.82	KEGG_PATHWAY	dre03010:Ribosome	40	8.15	11.13	1.72 × 10^−29^
	3	1	26.82	UP_KEYWORDS	Ribosomal protein	36	7.33	13.91	9.08 × 10^−28^
	3	1	26.82	GOTERM_MF_DIRECT	GO:0003735~structural constituent of ribosome	40	8.15	8.59	1.43 × 10^−22^
	3	1	26.82	UP_KEYWORDS	Ribonucleoprotein	37	7.54	9.3	5.58 × 10^−22^
	3	1	26.82	GOTERM_CC_DIRECT	GO:0030529~intracellular ribonucleoprotein complex	38	7.74	8.26	3.91 × 10^−21^
	3	2	3.55	GOTERM_CC_DIRECT	GO:0005852~eukaryotic translation initiation factor 3 complex	6	1.22	14.48	5.29 × 10^−3^
	3	2	3.55	GOTERM_CC_DIRECT	GO:0016282~eukaryotic 43S preinitiation complex	5	1.02	13.58	4.98 × 10^−2^
	3	3	3.41	GOTERM_CC_DIRECT	GO:0005852~eukaryotic translation initiation factor 3 complex	6	1.22	14.48	5.29 × 10^−3^
	4	2	1.98	PIR_SUPERFAMILY	PIRSF002285:Op18/stathmin	3	1.2	40.88	2.97 × 10^−2^
	5	1	8.29	INTERPRO	IPR008160:Collagen triple helix repeat	11	7.14	17.24	2.58 × 10^−7^
	5	1	8.29	UP_KEYWORDS	Collagen	9	5.84	20.41	1.06 × 10^−6^
	5	1	8.29	GOTERM_CC_DIRECT	GO:0005581~collagen trimer	9	5.84	20.13	5.32 × 10^−7^
	8	1	3.51	GOTERM_BP_DIRECT	GO:0019882~antigen processing and presentation	5	9.43	38.96	4.36 × 10^−4^
DEG significant at all timepoints	1	1	5.57	UP_KEYWORDS	Mitosis	6	10.34	38.18	2.83 × 10^−5^
	1	1	5.57	UP_KEYWORDS	Cell division	6	10.34	24.40	2.63 × 10^−4^
	1	1	5.57	GOTERM_BP_DIRECT	GO:0007067~mitotic nuclear division	6	10.34	20.46	1.69 × 10^−3^
	1	2	3.33	UP_KEYWORDS	Proteasome	4	6.90	29.22	2.00 × 10^−2^
	1	2	3.33	GOTERM_CC_DIRECT	GO:0000502~proteasome complex	4	6.90	25.14	2.07 × 10^−2^
	1	2	3.33	KEGG_PATHWAY	dre03050:Proteasome	4	6.90	21.84	1.04 × 10^−2^
	1	3	3.18	INTERPRO	IPR019821:Kinesin, motor region, conserved site	4	6.90	33.66	3.28 × 10^−2^
	1	3	3.18	SMART	SM00129:KISc	4	6.90	29.22	1.03 × 10^−2^
	1	3	3.18	INTERPRO	IPR001752:Kinesin, motor domain	4	6.90	29.22	4.96 × 10^−2^
	1	3	3.18	GOTERM_CC_DIRECT	GO:0005871~kinesin complex	4	6.90	23.43	2.54 × 10^−2^
	1	4	3.00	INTERPRO	IPR018525:Mini-chromosome maintenance, conserved site	3	5.17	129.05	3.40 × 10^−2^
	1	4	3.00	SMART	SM00350:MCM	3	5.17	113.97	9.46 × 10^−3^
	1	4	3.00	INTERPRO	IPR001208:Mini-chromosome maintenance, DNA-dependent ATPase	3	5.17	116.14	4.23 × 10^−2^
	1	4	3.00	GOTERM_CC_DIRECT	GO:0042555~MCM complex	3	5.17	94.26	1.76 × 10^−2^
	1	4	3.00	GOTERM_MF_DIRECT	GO:0003678~DNA helicase activity	3	5.17	84.78	3.33 × 10^−2^
	1	5	2.73	INTERPRO	IPR000243:Peptidase T1A, proteasome beta-subunit	3	5.17	116.14	4.23 × 10^−2^

## Data Availability

All data analyzed during this study are included in this article or Supplementary Materials. All raw data will be deposited in ArrayExpress.

## Supplementary Materials

The following supporting information can be downloaded at: https://www.mdpi.com/article/10.3390/cells11081373/s1, Figure S1: RNA-seq subset analysis gene clustering. Figure S2: DEG volcano plot. Differentially expressed genes at 3, 7 and 30 days compared to day 0. Table S1: Details of 1 and 2 antibodies. Table S2: Primary sequences and amplification temperatures for RT-PCR. Table S3: Differentially expressed genes. Table S4: Functional pathway annotation.

## Abbreviations

*ascl1a*	achaete–scute homolog 1
CMZ	ciliary marginal zone
CNS	central nervous system
*cxcr5*	C-X-C chemokine receptor type 5
dpi	days post injury
ECM	extracellular matrix
FC	fold change
FDR	false discovery rate
*gata3*	transcription factor GATA-3
GCL	ganglion cell layer
GFAP	glial fibrillary acidic protein
GFP	green fluorescent protein
H & E	hematoxylin and eosin
ILM	inner limiting membrane
INL	inner nuclear layer
IPL	inner plexiform layer
MG	Müller glia
*mmp2*	matrix metalloproteinase-2
*mmp9*	matrix metalloproteinase-9
OLM	outer limiting membrane
ONL	outer nuclear layer
OPL	outer plexiform layer
PBS	phosphate-buffered saline
PCNA	proliferating cell nuclear antigen
RPE	retinal pigment epithelium
RT-PCR	reverse transcriptase polymerase chain reaction
SEM	standard error of the mean
tgfbi	TGF-β induced
VST	variance stabilization transformation
*wnt4a*	wingless-type MMTV integration site family, member 4
