# Supplementary material for: Incomplete Recovery of Zebrafish Retina Following Cryoinjury
Source: Cells. 2022 Apr 18;11(8):1373. doi: 10.3390/cells11081373 (PMC9030934; doi:10.3390/cells11081373)
Supplement: Supplementary file 1 [file cells-11-01373-s001.zip › cells-1616709-Supplementary Material titles.pdf]

Figure S1. RNA-seq subset analysis gene clustering.

Genes showing differential expression over three time points scaled per-gene. A subset of top candidates with genome-wide significance expression FC at all three time points was assessed, identifying three distinct groups by hierarchical clustering. Table S4 lists significant associations in seven protein pathways. Analysis of all genes is shown in Figure 8. Fold change in gene expression is represented by color over days 3, 7, and 30; low – blue to high – red. Genes with common expression profiles were assessed for protein pathway enrichment. Query genes passing genome-wide significance at all time points is shown in Table S3, column 'FDRgenomeSignif'.

Figure S2. DEG volcano plot. Differentially expressed genes at 3, 7 and 30 days compared to day 0. A dotted line indicates significant threshold. Additionally, to prioritize genes with the strongest effect, DEG that had significant differences at all timepoints are shown in red. Obser-vation density shown at top by histogram. FDR sig all; significant DEG for genes at all timepoints (3, 7 and 30 dpi).

Table S1. Details of 1 and 2 antibodies.

Table S2. Primer sequences and amplification temperatures for RT-PCR.

Primer names, sequences, amplification temperature (Ta °C) and amplicon sizes (bp) specified for each primer pair used in RT-PCR gene expression analysis in Figure 7.

Table S3. Differentially expressed genes.

Scaled fold FC per-gene values for differentially expression genes and hierarchical clustering groups (column *clusterID*) as shown in Figure 8. Three measurement time points are included; days 3, 7, and 30. Average expression and gene annotation details are included. Genes passing genome-wide significance at all time points is shown in column 'FDRgenomeSignif'.

Table S4. Functional pathway annotation.

Differentially expressing genes were clustered into distinct expression profile groups (Table S3, Figure 8). Gene lists from each group were then used to query DAVID v6.8 as candidates of shared protein pathways. Significantly enriched pathways were identified using string selection criteria and Bonferroni correction for multiple testing. The column *Bonferroni* indicates corrected P-values with significant threshold <0.05. Sheet 1 lists summaries of (i) the differentially expressed gene cluster queried, (ii) number of query genes, (iii) number of matches in DAVID by ENSEMBL gene ID for organism *Danio rerio,* (iv) number of DAVID pathway clusters per query list, (v) number of significantly associated gene clusters. The full results for each set of query genes are listed in sheets 2-14. Genome wide significance at all time points is listed in sheet 15 'FDRgenomeSignif'.
