# Supplementary material for: Incomplete Recovery of Zebrafish Retina Following Cryoinjury
Source: Cells. 2022 Apr 18;11(8):1373. doi: 10.3390/cells11081373 (PMC9030934; doi:10.3390/cells11081373)
Supplement: Supplementary file 1 [file cells-11-01373-s001.zip › Table S1.pdf]

Table S1 Details of 1° and 2° antibodies.

1° ab				
Antibody	Species	Isotype	Dilution	Product Code & Source
anti-PCNA	rabbit, polyclonal	IgG	1:400	ab2426, Abcam
zpr-1	mouse, monoclonal	IgG	1:500	AB_10013803, ZIRC
zpr-3	mouse, monoclonal	IgG	1:100	AB_10013805, ZIRC
zns-2	mouse, monoclonal	IgG	1:400	AB_10013793, ZIRC
zrf-1	mouse, monoclonal	IgG	1:400	AB_10013806, ZIRC
2° ab				
Antibody	Species	Isotype	Dilution	Product Code & Source
Alexa Fluor 488	goat anti-rabbit	IgG	1:500	A11008, Invitrogen
Alexa Fluor 488	goat anti-mouse	IgG	1:500	A11017, Invitrogen
Alexa Fluor 594	goat anti-mouse	IgG	1:500	A11005, Invitrogen
Alexa Fluor 594	goat anti-rabbit	IgG	1:500	A11012, Invitrogen
