# Supplementary material for: Incomplete Recovery of Zebrafish Retina Following Cryoinjury
Source: Cells. 2022 Apr 18;11(8):1373. doi: 10.3390/cells11081373 (PMC9030934; doi:10.3390/cells11081373)
Supplement: Supplementary file 1 [file cells-11-01373-s001.zip › Table S2.pdf]

Table S2 Primer sequences and amplification temperatures for RT-PCR.

Gene ID	Direction	Sequence 5’– 3’	Ta °C	Amplicon size (bp)
ascl1a	Forward	GCTCCTGGACTTCACCAACT	58/60	230
	Reverse	TGCGCTGGAAGGACTGGATT		
lin28	Forward	GACTCACCCGTGGATGTCTT	60	207
	Reverse	ATCTCCTTTTGACCGCCTCT		
wnt4a	Forward	CTGGAGAGCGTTCCTGTGTT	60	251
	Reverse	CGTTCCCTGATGTCCACGAA		
mmp9	Forward	GCCGTATCTCTGTTAGGGCA	60	199
	Reverse	GCTTCTGTCCCAGTGAGCTT		
mmp2	Forward	CCACCAGTCACACCAATGGA	60	244
	Reverse	CCCACATCTCATCACCAGCA		
tgfbi	Forward	ATCAGGCGGTTCTTCAACAC	60	127
	Reverse	TTGCGGCGATACCATTGCTT		
cxcr5	Forward	TTCCTGTGCAAGCTAGTGGG	60	164
	Reverse	CAGAGCAGTCCACAGGTGAG		
gata3	Forward	CTTGAAGCCTCGCACTGATG	60	185
	Reverse	ACAGCTCGATTCCTCGTGTC		
stat3	Forward	AGCAGTTGGAGACGCGGTAT	58	138
	Reverse	TGGCGTGAGACTCCTTGTTG		
