# Supplementary figures and images for: Incomplete Recovery of Zebrafish Retina Following Cryoinjury
Source: Cells. 2022 Apr 18;11(8):1373. doi: 10.3390/cells11081373 (PMC9030934; doi:10.3390/cells11081373)

Figure S1 RNA-seq subset analysis gene clustering.

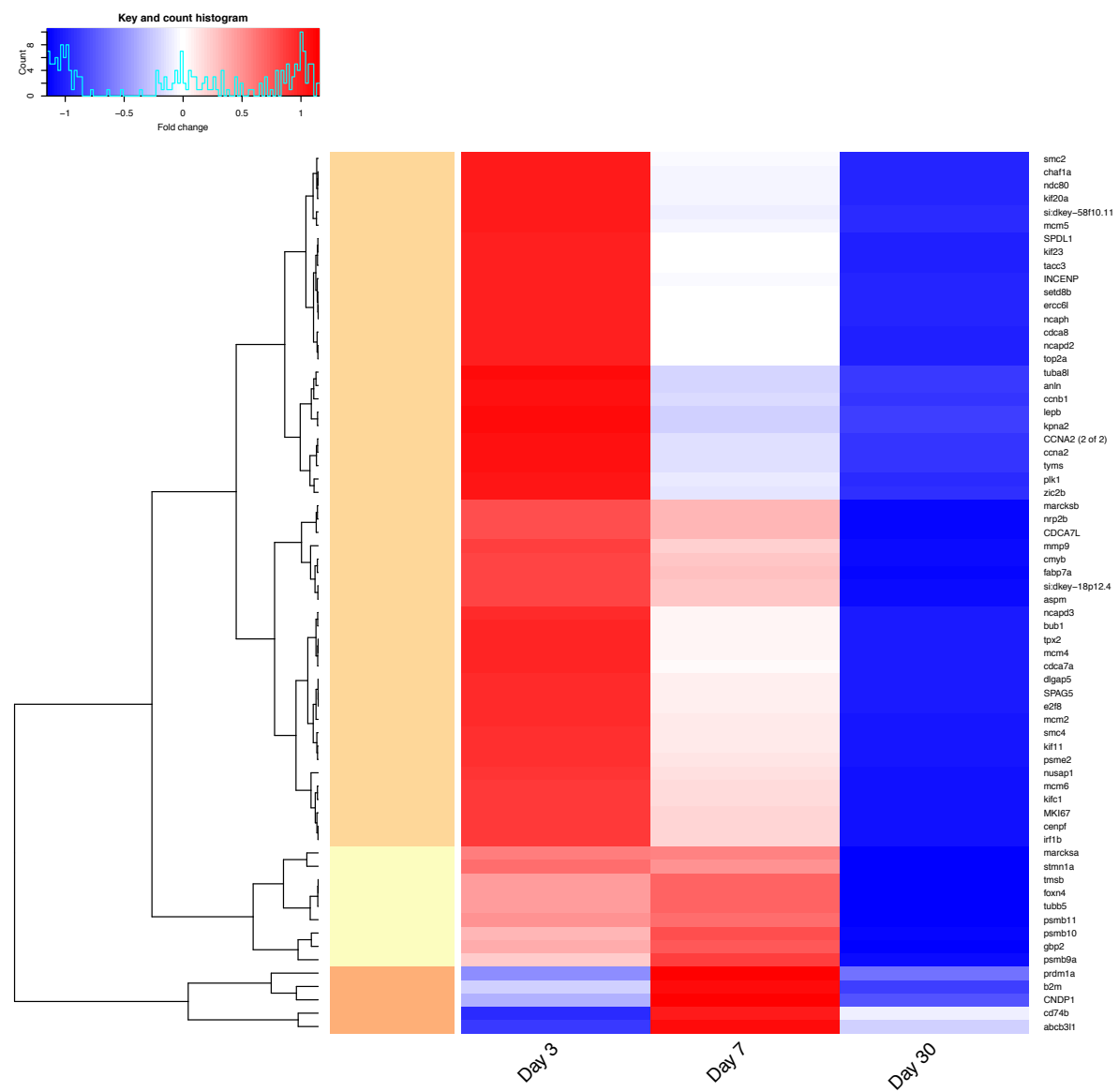

Supplement: Supplementary file 1 [file cells-11-01373-s001.zip › Figure S1.pdf]

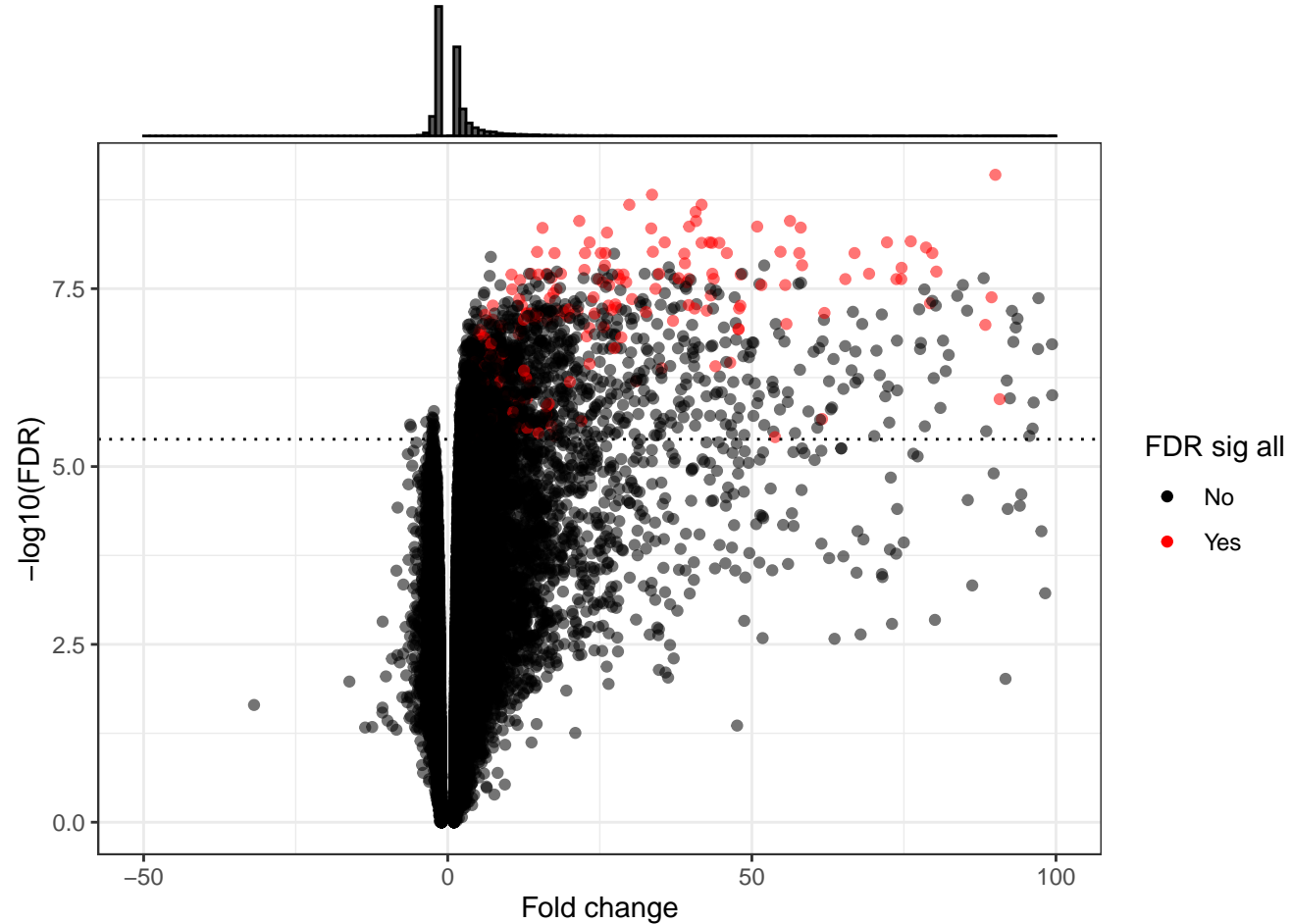

Supplement: Supplementary file 1 [file cells-11-01373-s001.zip › Figure S2.pdf]
